# Ketamine for Bipolar Depression: A Systematic Review

**DOI:** 10.1093/ijnp/pyab023

**Published:** 2021-04-30

**Authors:** Anees Bahji, Carlos A Zarate, Gustavo H Vazquez

**Affiliations:** 1 Department of Psychiatry, University of Calgary, Calgary, Alberta, Canada; British Columbia Centre for Substance Use, Vancouver, British Columbia, Canada; Research in Addiction Medicine Scholars [RAMS] Program, Boston University Medical Centre, Boston, MA, USA; 2 Section Neurobiology and Treatment of Mood Disorders, Division of Intramural Research Program, National Institute of Mental Health, Bethesda, Maryland, USA; 3 Department of Psychiatry, Queen’s University, Kingston, Ontario, Canada

**Keywords:** Bipolar disorder, depression, ketamine, systematic review

## Abstract

**Background:**

Ketamine appears to have a therapeutic role in certain mental disorders, most notably unipolar major depressive disorder. However, its efficacy in bipolar depression is less clear. This study aimed to assess the efficacy and tolerability of ketamine for bipolar depression.

**Methods:**

We conducted a systematic review of experimental studies using ketamine for the treatment of bipolar depression. We searched PubMed, MEDLINE, Embase, PsycINFO, and the Cochrane Central Register for relevant studies published since each database’s inception. We synthesized evidence regarding efficacy (improvement in depression rating scores) and tolerability (adverse events, dissociation, dropouts) across studies.

**Results:**

We identified 6 studies, with 135 participants (53% female; 44.7 years; standard deviation, 11.7 years). All studies used 0.5 mg/kg of add-on intravenous racemic ketamine, with the number of doses ranging from 1 to 6; all participants continued a mood-stabilizing agent. The overall proportion achieving a response (defined as those having a reduction in their baseline depression severity of at least 50%) was 61% for those receiving ketamine and 5% for those receiving a placebo. The overall response rates varied from 52% to 80% across studies. Ketamine was reasonably well tolerated; however, 2 participants (1 receiving ketamine and 1 receiving placebo) developed manic symptoms. Some participants developed significant dissociative symptoms at the 40-minute mark following ketamine infusion in 2 trials.

**Conclusions:**

There is some preliminary evidence supporting use of intravenous racemic ketamine to treat adults with bipolar depression. There is a need for additional studies exploring longer-term outcomes and alterative formulations of ketamine.

## Introduction

Bipolar depression is a leading cause of disability globally, affecting nearly 1% of individuals worldwide (Ferrari et al., 2016). As in unipolar depression, treatment-resistant bipolar depression (TRBD) is widespread but remains understudied (Sachs, 1996; Gitlin, 2006; Sienaert et al., 2013). One definition of TRBD involves the failure to reach sustained symptomatic remission for 8 consecutive weeks after 2 different treatment trials, at adequate therapeutic doses, with at least 2 recommended monotherapy treatments or at least 1 monotherapy treatment and another combination treatment (Hidalgo-Mazzei et al., 2019).

Despite the importance of TRBD, only a small number of recognized treatment options are available (Hidalgo-Mazzei et al., 2019). A few trials have indicated a role for electroconvulsive therapy and repetitive transcranial magnetic stimulation (Schoeyen et al., 2015; Tavares et al., 2017). While recent network meta-analyses have shown consistent evidence for use of multiple pharmacotherapies in non-TRBD (Bahji et al., 2020a, 2020b, 2021a, 2021c), there is more limited evidence for use of medication-based treatments in TRBD.

Fortunately, there appears to be an emerging role for ketamine in managing unipolar depression (McIntyre et al., 2020; Bahji et al., 2021b, 2021c). Early ketamine studies demonstrated rapid, potent reductions in depressive symptoms following administration of a single sub-anesthetic dose of intravenous racemic ketamine (Berman et al., 2000; Zarate et al., 2006; Ionescu et al., 2015; Hu et al., 2016; Wilkinson et al., 2018). While these initial results were promising, effective means of maintaining the acute effects were actively sought (Phillips et al., 2019). To date, the use of other glutamatergic agents to prolong the acute antidepressant effects of ketamine has been mostly inconsistent, with some successful case reports and small open-label studies (Zarate et al., 2005; Mathew et al., 2010; Ibrahim et al., 2012; Caddy et al., 2015; McCloud et al., 2015). While repeat doses of intravenous racemic ketamine appear to sustain short-term antidepressant effects for individuals with unipolar depression (Murrough et al., 2013; Ghasemi et al., 2014; López-Díaz et al., 2017; Ionescu et al., 2019), it is unclear whether this holds for bipolar depression. Racemic ketamine can also rapidly reduce suicidal thoughts within 1 day and for up to 1 week in depressed patients with suicidal ideation (Reinstatler and Youssef, 2015; López-Díaz et al., 2017; Grunebaum et al., 2018; Wilkinson et al., 2018; Williams et al., 2019; Witt et al., 2020). While these findings are mostly limited to unipolar depression, some emerging studies point to the efficacy of ketamine for bipolar significant depression (Zarate et al., 2012; Grunebaum et al., 2017; Chen et al., 2019). Racemic ketamine has also led to many preclinical and biomarker discoveries (Zanos et al., 2016; Zanos and Gould, 2018), leading to new possibilities and safer alternatives for mitigating dissociation and reducing the propensity for misuse and diversion of ketamine (Newport et al., 2015; Burger et al., 2016; Lener et al., 2017).

Although clinical studies of ketamine for TRBD are now underway, the level of proof of efficacy remains low, and more RCTs are needed to explore efficacy and safety issues of ketamine (Corriger and Pickering, 2019). While previous reviews have explored ketamine’s utility in the treatment of TRBD, there is a need to update previous reviews given the recent increase in ketamine studies.

## OBJECTIVE

We aimed to provide an updated synthesis of findings from studies examining the efficacy and safety of ketamine for bipolar depression.

## METHODS

### Protocol and Registration

We registered this study with the Open Science Framework (https://osf.io/ksvnb/). We followed the Preferred Reporting Items for Systematic Reviews and Meta-analyses (Liberati et al., 2009).

### Eligibility Criteria

We included randomized controlled trials and nonrandomized studies examining the use of ketamine in adults (aged 18 years or older) to treat bipolar depression. We considered studies examining any formulation of ketamine (e.g., intravenous racemic ketamine, intranasal enantiomeric *S*-ketamine [esketamine]) as a standalone treatment or in combination with psychotropic medications or psychotherapies. We excluded observational designs (i.e., surveys, cohort studies, case series, and case-control studies), reviews, post hoc and secondary analyses, commentaries, and clinical overviews. We also excluded studies pairing ketamine with a neurostimulation-based treatment. We only included studies reporting at least 1 outcome related to the efficacy or safety of ketamine, such as the response to treatment or adverse events. Finally, we excluded studies that did not separate participants with bipolar depression from those with unipolar depression (Berman et al., 2000).

### Information Sources and Search

We searched MEDLINE, Embase, PsycINFO, the Cochrane Central Register of Controlled Clinical Trials (CENTRAL), and the Cochrane Database of Systematic Reviews via Ovid for studies published from inception to December 13, 2019. To identify ongoing or unpublished studies, we also searched ClinicalTrials.gov, the EU Clinical Trials Register, and the Australian and New Zealand Clinical Trials Registry using the keywords “ketamine” and “bipolar depression.” We also hand-searched reference lists of included studies and topical reviews for potentially relevant articles.

### Study Selection

Two researchers (AB, GHV) independently examined titles and abstracts using the web-based systematic review program Covidence (Veritas Health Innovation, 2019). Relevant articles were obtained in full and assessed for inclusion independently by the 2 coauthors. Any disagreement between them was resolved via discussion to reach a consensus.

### Data Collection Process and Data Items

Two co-authors (AB, GHV) extracted data via a pre-piloted, standardized data extraction tool in Microsoft Excel 2016. We pulled data on details of the populations, interventions, comparisons, outcomes of significance to the mental disorder, study methods, ketamine dose and route of administration, study withdrawals, and study withdrawals due to adverse events. Where data were missing, we contacted the authors for additional information. When authors reported multiple analyses (e.g., intention-to-treat or per-protocol analyses), we extracted the more conservative analysis, with a preference for intention-to-treat analyses.

### Risk of Bias in Individual Studies

We assessed the risk of bias within individual trials using the Cochrane risk of bias tool for randomized controlled trials. Specifically, the bias tool assesses indicators of selection bias, performance bias, detection bias, attrition bias, and reporting bias (Higgins et al., 2011). The risk of bias assessments were completed independently by 2 authors (AB or GHV). Inter-researcher disagreements were resolved via discussion to reach a consensus.

### Analytic Methods

While we intended to conduct a meta-analysis, we only identified a total of 6 studies, of which only 3 were randomized controlled trials. Instead, we present the results in tables and discuss the findings comprehensively in the text.

## RESULTS

### Study Selection

**Figure 1.**
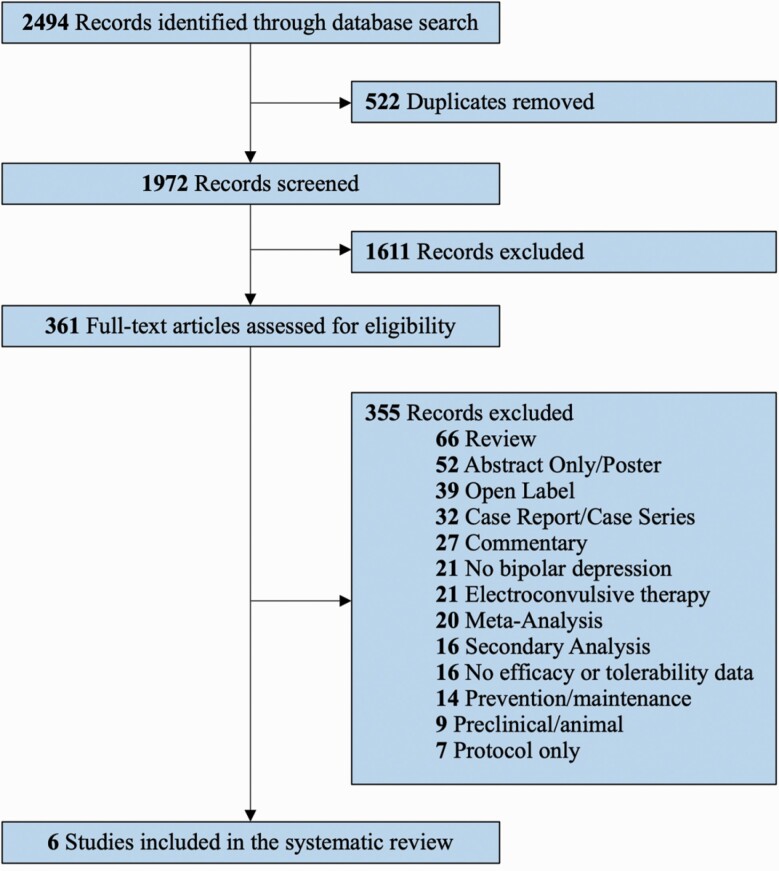
PRISMA flow diagram outlining the systematic review process. Abbreviation: PRISMA, Preferred Reporting Items for Systematic Reviews and Meta-Analyses.

The search strategy identified a total of 2494 records (Figure 1). After removing duplicates, we screened the remaining 1972 unique articles by title and abstract. We then excluded 1611 irrelevant records, leaving 361 documents for a full-text review. After a full-text review, only 6 studies met the final inclusion criteria (Diazgranados et al., 2010; Zarate et al., 2012; Rybakowski et al., 2013; Permoda-Osip et al., 2014; Grunebaum et al., 2017; Zheng et al., 2020).

### Characteristics of Studies, Participants, and Interventions

Table 1 provides an overview of the study characteristics. Three studies were randomized controlled trials (Diazgranados et al., 2010; Zarate et al., 2012; Grunebaum et al., 2017), while the other 3 were open-label, single-arm studies (Rybakowski et al., 2013; Permoda-Osip et al., 2014; Zheng et al., 2020). By country, most studies were from the United States (50%; k = 3) or Poland (33%; k = 2). There were 135 participants (53% female; 44.7 years; standard deviation, 11.7 years). Except for 1 study not reporting the diagnostic criteria for bipolar depression (BD; Permoda-Osip et al., 2014), the remaining 5 used Diagnostic and Statistical Manual of Mental Disorders fourth or fifth edition criteria. All 6 studies used add-on racemic ketamine at a dose of 0.5 mg/kg delivered intravenously; hence, all participants continued treatment with a primary mood-stabilizing agent throughout ketamine treatment. However, the number of doses varied across studies, with 3 single-dose studies (Rybakowski et al., 2013; Permoda-Osip et al., 2014; Grunebaum et al., 2017), 2 studies with 2 test doses (1 ketamine and 1 placebo) 2 weeks apart (Diazgranados et al., 2010; Zarate et al., 2012), and 1 study with 6 doses across 2 weeks (Zheng et al., 2020). Except for 1 study (Grunebaum et al., 2017), the remaining 5 involved TRBD, defined as an insufficient response to at least 1 (Rybakowski et al., 2013; Permoda-Osip et al., 2014) or 2 (Diazgranados et al., 2010; Zarate et al., 2012; Zheng et al., 2020) previous antidepressant trials. Two trials also required that participants have an inadequate response to prospective trials of lithium or valproate (Diazgranados et al., 2010; Zarate et al., 2012). Notably, most excluded those who had cooccurring general medical conditions, were pregnant or breastfeeding, or had comorbid psychosis or addiction.

**Table 1.** Study characteristics (k = 6)

Study	Design	Population	Intervention(s)	Primary Findings
Diazgranados et al., 2010	Crossover RCT	TRBD (n = 17)	Racemic, adjunctive ketamine: 0.5 mg/ kg IV on 2 test days 2 weeks apart vs. placebo	71% (vs. 6%) responded to ketamine (vs. placebo) during the trial on the MADRS. Dissociative symptoms occurred at the 40-minute mark. One participant in each group developed manic symptoms.
Zarate et al., 2012	Crossover RCT	TRBD (n = 15)	Racemic, adjunctive ketamine: 0.5 mg/ kg IV on 2 test days 2 weeks apart vs. placebo	79% (vs. 0%) responded to ketamine (vs. placebo) during the trial on the MADRS. Dissociative symptoms occurred at the 40-minute mark.
Rybakowski et al., 2013	Open-label, single-arm trial	TRBD (n = 25)	Racemic, adjunctive ketamine: 0.5 mg/ kg IV, single dose	Using the HDRS, there were 13 ketamine responders and 8 remitters after 7 and 14 days, respectively. Serum NGF, NT3, NT4, and GDNF did not significantly change.
Permoda-Osip et al., 2014	Open-label, single-arm trial	TRBD (n = 42)	Racemic, adjunctive ketamine: 0.5 mg/ kg IV, single dose	HDRS scores reduced significantly after 24 hours (from 22.6±5.1 hours to 15.6±7.4 hours), 7 days (to 13±7 days), and 14 days (to 11.8±7.8 days).
Grunebaum et al., 2017	Parallel RCT	Non-TRBD (n = 16)	Racemic, adjunctive ketamine: 0.5 mg/ kg IV, single dose vs. midazolam 0.02 mg/kg IV	HDRS and SSI scores reduced by approximately 6 points each in the ketamine group, but the differences were not statistically significant.
Zheng et al., 2020	Open-label, single-arm trial	TRBD (n = 19)	Racemic, adjunctive ketamine: 0.5 mg/ kg IV, 6 doses over 12 days	Rates of response and remission were 73.7% and 63.2% at the study end. There were no significant dissociative and psychotomimetic symptoms on the CADSS or BPRS.

Abbreviations: BPRS, Brief Psychiatric Rating Scale; CADSS, Clinician-Administered Dissociative States Scale; GDNF, glial-derived neurotrophic factor; HDRS, Hamilton Depression Rating Scale; IV, intravenous; MADRS, Montgomery-Åsberg Depression Rating Scale; NGF, nerve growth factor; NTF3, neurotrophin-3; NTF4, neurotrophin-4; RCT, randomized controlled trial; SSI, Scale for Suicidal Ideation; TRBD, treatment-resistant bipolar depression.

### Efficacy of Intravenous, Racemic Ketamine for Bipolar Depression

Across all 6 studies, the proportion achieving a response (defined as those having a reduction in their baseline depression severity of at least 50%) was 61% for those receiving ketamine at some point during the trial (77/126). The overall response rate across studies varied from 52% (Rybakowski et al., 2013) to 80% (Zarate et al., 2012). For the 3 studies that involved control groups, the overall pooled response rate was only 5% (2/42). There were improvements in depression rating scores over time in all studies; however, in the 1 trial using a midazolam control in non-TRBD subjects, the difference was not statistically significant (Grunebaum et al., 2017). The efficacy of single-dose ketamine did not extend beyond the 2-week mark; however, the study that used 6 doses of ketamine over 2 weeks appeared to show longer-lasting efficacy.

### Tolerability of Intravenous, Racemic Ketamine for Bipolar Depression

Across most of the included studies, participants tolerated ketamine treatment reasonably well. However, there were some significant adverse events. For example, 2 participants (1 receiving ketamine and 1 receiving placebo) developed manic symptoms (Diazgranados et al., 2010). In 2 trials, participants developed significant dissociative symptoms, primarily at the 40-minute mark following ketamine infusion (Diazgranados et al., 2010; Zarate et al., 2012). However, the remaining 4 trials did not note substantial dissociation or mania symptoms at any point during the study.

### Study Quality and Risk of Bias

Three studies were double-blind, randomized, controlled trials with concealed allocation (Diazgranados et al., 2010; Zarate et al., 2012; Grunebaum et al., 2017). These studies were at very low risk of bias as per the Cochrane Risk of Bias Tool. The remaining 3 were all nonrandomized, open-label, single-arm studies, which lacked a control group and were more susceptible to participation bias.

## Discussion

To our knowledge, this is the most recent systematic review that has explored the effectiveness and tolerability of ketamine for the treatment of BD. Overall, our findings—derived from 6 studies—indicate that ketamine appears to be an effective and relatively safe treatment for BD and TRBD.

All 6 studies in our review involved intravenous racemic ketamine at a dose of 0.5 mg/kg as an add-on treatment to primary mood-stabilizing medications. To that end, the rapid antidepressant effects of ketamine seen in individuals with TRBD appears to be predictive of a sustained outcome (Murrough et al., 2011, 2013; Atigari and Healy, 2013; Ionescu et al., 2014).

In a previous meta-analysis, there was no significant difference in the clinical response to intravenous ketamine between patients with unipolar major depression and bipolar depression (Bahji et al., 2021c). However, there are no available studies on intranasal esketamine for bipolar depression; hence, there are still unclear aspects concerning the role of ketamine in bipolar disorder. In contrast, several prior studies indicate a role for intravenous ketamine in treating bipolar depression (Ionescu et al., 2015; Bobo et al., 2016; Alberich et al., 2017; Kraus et al., 2017; López-Díaz et al., 2017; Gałuszko-Węgielnik et al., 2019). For very short-term use, the available data demonstrates a clear and consistent antidepressive effect of ketamine versus esketamine treatment in unipolar major depression, relative to various control conditions, beginning within hours of administration and lasting up to 7 days after a single dose (McGirr et al., 2015; Bahji et al., 2021c). However, we do not know whether this pattern is also present in cases of bipolar disorder, where we only have data for racemic ketamine. Hence, there is a need for head-to-head studies comparing ketamine to esketamine in bipolar disorder. Future studies could also measure blood levels of ketamine and norketamine with intravenous racemic ketamine and esketamine and determine whether the differences remain significant after controlling.

Regarding the side effect profiles, most studies indicate that ketamine is reasonably well tolerated for bipolar depression treatment. Two significant concerns involve the risk of dissociation and induction of mania or hypomania. However, in our review, most trials and most participants did not experience either of these adverse events. However, in 2 trials, participants developed significant dissociative symptoms, primarily at the 40-minute mark following ketamine infusion (Diazgranados et al., 2010; Zarate et al., 2012). However, the remaining 4 trials did not note substantial dissociation or mania symptoms at any point during the study. A related concern for ketamine in bipolar disorder involves the risk of switching to a manic or hypomanic episode. In 1 trial, 2 participants (1 receiving ketamine and 1 receiving placebo) developed manic symptoms (Diazgranados et al., 2010). While standard antidepressants can induce rapid cycling, it is unclear whether this can occur with ketamine, as trials are typically short. Still, mania switches with single-ketamine infusions or pulsed treatment (where repeated doses are spaced over several days or weeks) have had small sample sizes, which may be insufficiently powered to identify manic switching. However, there was insufficient evidence to support mania induction with a single subanesthetic dose of ketamine in 98 major depressed patients (Niciu et al., 2013).

Nonetheless, there is a real necessity in our therapeutic armamentarium to discover and add more effective and safer treatments for patients with TRBD (Gao et al., 2016). Part of the challenge in elucidating the comparative performance of different formulations of ketamine may lie in the lack of a clear consensus on the mechanisms underlying ketamine’s therapeutic effects (Strasburger et al., 2017; Zanos and Gould, 2018). With the isolation of esketamine, there was also an option of providing much lower doses of ketamine and the opportunity to reduce the dose-dependent dissociative properties of ketamine (Correia-Melo et al., 2018). As esketamine was also available through an intranasal delivery system, it presented a substantially more practical option than intravenous racemic ketamine (Tibensky et al., 2016; Schatzberg, 2019). Ultimately, intranasal esketamine was approved by the US Food and Drug Administration on March 5, 2019, for use in TRBD (Kim et al., 2019); on December 19, 2019, Europe followed suit with approval of esketamine for the same indication (Wei et al., 2020).

### Limitations

Although this review has several strengths, a few fundamental limitations deserve some expansion here. First, we were unable to conduct a meta-analysis given the low study yield. Despite this, we were still able to present the results across studies in a meaningful way. Second, there is always the possibility of publication bias, such that our review may not have identified negative studies (where ketamine did not improve depression scores). Third, while our review attempted to cover as much follow-up time as possible after ketamine treatment administration, there remains minimal information regarding longer-term follow-up beyond the 2-week mark. Hence, the results of our study are also limited to this treatment window. Fourth, participants in the presented trials are relatively unrepresentative of the real-world population with BD, given the studies’ strict exclusion criteria. Thus, the results of the trials may not represent the real-world efficacy of ketamine. Fifth, the high heterogeneity within the selected studies could have impacted our findings. For example, there are differences between patients with TRBD versus non-TRBD, between those who receive treatment as an inpatient versus at community clinics, and between studies where participants received single or multiple ketamine doses.

## Conclusions

Ketamine represents an innovative, rapidly acting, experimental treatment for bipolar depression. This review found that preliminary evidence supports the use of intravenous racemic ketamine for the treatment of individuals with bipolar depression. At present, there are no studies that have used other formulations of ketamine, such as intranasal esketamine. To that end, the intravenous administration route presents a practical limitation. Future studies should explore the use of an intranasal formulation of esketamine for individuals with bipolar depression. While racemic ketamine has demonstrated significant short-term benefits in several clinical studies highlighted in this review, the long-term benefits remain insufficiently explored, which may contribute to the current lack of Food and Drug Administration approval for use in individuals with bipolar disorder. At present, the level of proof of efficacy remains low. More randomized controlled trials are needed to explore efficacy and safety issues for administering all forms of ketamine in the treatment of bipolar depression.

## Statement of Interest

C.A.Z. is listed as a coinventor on a patent for the use of (2R,6R)-HNK and other hydroxynorketamines in the treatment of depression. C.A.Z. is listed as a coinventor on a patent application for the use of (2R,6R)-HNK and (2S,6S)-HNK in the treatment of depression, anxiety, anhedonia, suicidal ideation, and post-traumatic stress disorder. C.A.Z. is listed as a coinventor on a patent application for the crystal forms and synthesis of (2R,6R)-hydroxynorketamine and (2S,6S)-hydroxynorketamine.
